# International Consensus Statements on Endoscopic Ultrasound‐Guided Biliary Drainage: Results From the 1st JGES International in 2025

**DOI:** 10.1111/den.70194

**Published:** 2026-07-10

**Authors:** Takuji Iwashita, Shuntaro Mukai, Yousuke Nakai, Shomei Ryozawa, Akio Katanuma, Ichiro Yasuda, Atsushi Irisawa, Sang‐Soo Lee, Jong Ho Moon, Hsui‐Po Wang, Sundeep Lakhtakia, Marc Giovannini, Todd H. Baron, Takao Itoi

**Affiliations:** ^1^ Department of Gastroenterolog Shiga University of Medical Science Otsu Shiga Japan; ^2^ Tokyo Medical University Tokyo Japan; ^3^ Department of Internal Medicine, Institute of Gastroenterology Tokyo Women's Medical University Tokyo Japan; ^4^ Department of Gastroenterology Saitama Medical University International Medical Center Saitama Japan; ^5^ Department of Gastroenterology Sapporo Medical University Sapporo Hokkaido Japan; ^6^ Third Department of Internal Medicine University of Toyama Toyama Japan; ^7^ Department of Gastroenterology Dokkyo Medical University School of Medicine Shimotsuga Tochigi Japan; ^8^ Department of Gastroenterology Good Gang‐An Hospital Busan Korea; ^9^ Department of Internal Medicine SoonChunHyang University College of Medicine Bucheon Korea; ^10^ Department of Internal Medicine, National Taiwan University Hospital National Taiwan University College of Medicine Taipei Taiwan; ^11^ Department of Gastroenterology AIG Hospitals Hyderabad India; ^12^ Department of Gastroenterology IRCAD Strasbourg France; ^13^ Department of Gastroenterology and Hepatology University of North Carolina Chapel Hill North Carolina USA

**Keywords:** antegrade treatment, choledochoduodenostomy, hepaticogastrostomy, surgically altered anatomy, terminology

## Abstract

EUS‐guided biliary drainage (EUS‐BD) has evolved as an alternative to ERCP‐BD for biliary obstruction, but its role as a primary drainage and its terminology have not been standardized.

**Aims:**

To establish an international consensus on the indications, techniques, and standardized terminology of EUS‐BD.

**Methods:**

To address these, an international consensus meeting was held during the 1st JGES International. Fourteen expert endoscopists from six countries participated in a structured Delphi process, reviewing evidence and voting on clinical questions related to EUS‐BD indications, techniques, and terminology, particularly for unresectable malignant distal biliary obstruction (MDBO) and for biliary diseases in patients with surgically altered anatomy (SAA).

**Results:**

For unresectable MDBO, EUS‐BD was recognized as a safe and effective alternative to ERCP‐BD, although it is not yet established as a standard first‐line approach. It is an established salvage option after failed ERCP and may be considered as an upfront option in selected patients such as those with duodenal obstruction preventing access to the papilla. The choice between choledochoduodenostomy and hepaticogastrostomy varies among countries depending on device availability and expertise. In patients with SAA, EUS‐BD showed higher technical success and shorter procedure time than balloon endoscopy–assisted ERCP, though either approach may be selected based on institutional and endoscopist experience. A unified terminology for interventional EUS is also proposed, including the concepts of anastomosis versus drainage and trans‐ESCR (endosonographically created route) procedures.

**Conclusions:**

This consensus highlights the need for standardized global terminology and further trials to define the optimal role of EUS‐BD for biliary diseases.

**Abbreviations**
AG: antegrade technique
BBS: benign biliary stricture
BDS: bile duct stones
BE: balloon endoscopy
BE‐ERCP: balloon endoscopy‐assisted ERCP
CDS: choledochoduodenostomy
EDGE: EUS‐directed transgastric ERCP
ERCP‐BD: endoscopic retrograde cholangiopancreatography‐guided biliary drainage
ESCR: endosonographically created route
EUS‐BD: endoscopic ultrasound‐guided biliary drainage
FCMS: fully covered metallic stent
GBD: gallbladder drainage
HGS: hepaticogastrostomy
JGES: Japan Gastroenterological Endoscopy Society
LAMS: lumen‐apposing metal stent
MBO: malignant biliary obstruction
MDBO: malignant distal biliary obstruction
PCMS: partially covered metallic stent
PS: plastic stent
PTBD: percutaneous transhepatic biliary drainage
RCT: randomized controlled trial
SAA: surgically altered anatomy
T‐DAS: transluminal drainage/anastomosis stent

## 1 Background

Since its first report in 2001 [1], endoscopic ultrasound‐guided biliary drainage (EUS‐BD) has evolved into a reliable salvage procedure for failed or difficult endoscopic retrograde cholangiopancreatography‐guided biliary drainage (ERCP‐BD). EUS‐BD encompasses several approaches, including transmural biliary drainage, such as choledochoduodenostomy (CDS) and hepaticogastrostomy (HGS); the rendezvous technique for difficult biliary cannulation; and the antegrade (AG) technique for managing biliary diseases through an EUS‐created tract. With the advancement of dedicated devices and techniques, EUS‐BD has been increasingly recognized as an effective and safe alternative to percutaneous drainage [2]. However, its role as a primary drainage modality, particularly for unresectable malignant distal biliary obstruction (MDBO) and biliary diseases in surgically altered anatomy (SAA), remains under discussion [3, 4, 5].

Another issue associated with the rapid expansion of interventional EUS, including EUS‐BD, is the lack of standardized terminology and classification. Although EUS‐BD has rapidly evolved and become increasingly incorporated into routine clinical practice, its terminology and classification have not previously been systematically discussed. Consequently, individual researchers have adopted their own definitions and nomenclature. To address this issue, a subcommittee for terminology of interventional endoscopic ultrasonography/endosonography that was established within the Terminology Committee of the Japan Gastroenterological Endoscopy Society (JGES) proposed a standardized framework entitled “Proposal of Classification and Terminology of Interventional Endoscopic Ultrasonography/Endosonography” [6].

To address these unresolved issues, harmonize global understanding of EUS‐BD, and share the proposed classification and terminology, an international consensus meeting was convened during the 1st JGES International—the official international congress of the Japan Gastroenterological Endoscopy Society (JGES)—held on September 5–6, 2025, in Tokyo, Japan. This JGES International Consensus Meeting was conducted with financial support from JGES. The meeting aimed to discuss the current status, indications, techniques, and future directions of EUS‐BD.

## 2 Methods

This consensus meeting was organized by a panel of endoscopists from multiple high‐volume centers worldwide. Experts were invited based on their extensive experience in pancreatobiliary endoscopy, including both ERCP and EUS‐guided interventional procedures. Ultimately, 14 expert endoscopists from six countries participated in the consensus process.

A structured Delphi method was employed to establish agreement. In this process, all designated panelists independently reviewed the available evidence and provided feedback on each proposed statement through a series of iterative rounds. Levels of agreement were graded as “strongly agree,” “partially agree,” “partially disagree,” or “disagree.” For each question, a minimum of 80% agreement among panelists was required to reach consensus. Statements not meeting this threshold were revised or excluded after further discussion. At least two iterative rounds of voting were conducted under the supervision of the lead coordinator.

Following brief presentations summarizing the current evidence on EUS‐BD—specifically its role as a primary drainage option for unresectable MDBO and its application for biliary diseases (stones and MDBO) in patients with surgically altered anatomy (SAA)—the panel discussed key clinical questions and formulated consensus statements based on the Delphi process. Finally, an open discussion was held to refine the Japan Gastroenterological Endoscopy Society's proposed standardized classification and terminology for interventional EUS, ensuring that the terminology appropriately reflected current EUS‐BD practices among international experts.

## 3 Clinical Questions and Consensus Statements

### 3.1 Topic 1: EUS‐BD as a Primary Drainage Option in the Management of Unresectable MDBO

MDBO has traditionally been managed by ERCP‐BD, and EUS‐BD is performed as a salvage procedure for failed or difficult ERCP‐BD cases. However, there have been several randomized controlled trials (RCTs) that compared the efficacy and safety of EUS‐BD with ERCP‐BD for MDBO as primary biliary drainage. To date, five RCTs comparing EUS‐BD with ERCP‐BD have been reported [7, 8, 9, 10, 11]. The study outcomes are summarized in Table 1. Across these studies, technical success rates of EUS‐BD were equal to or higher than those of ERCP‐BD, with significantly greater success observed in certain trials, while clinical success rates were uniformly high in both groups, generally exceeding 90%. In terms of safety, the overall incidence of adverse events was similar to or lower than that with EUS‐BD, and notably, post‐ERCP pancreatitis was virtually absent. Procedure time was also consistently shorter for EUS‐BD than for ERCP‐BD, reflecting greater procedural efficiency. Taken together, these findings indicate that EUS‐BD appears to be not only a noninferior but also a promising alternative to ERCP for primary biliary drainage in MDBO and may be considered a safe, effective, and increasingly accepted approach.

**TABLE 1:** Randomized controlled studies comparing EUS‐BD versus ERCP‐BD for MDBO as primary drainage.

Study (author, year)	Subject	Details of EUS‐BD	EUS‐BD versus ERCP‐BD					
			Number of patients, *n*	Technical success, %	Clinical success, %	Adverse event, %	PEP (%)	Procedure time, min
Bang et al., 2018	MBO by PC	CDS using SEMS	33 vs. 34	90.9 vs. 94.1	97.0 vs. 91.2	21.2 vs. 14.7	0 vs. 2.9	25 vs. 21 (median)
Paik et al., 2018	Unresectable MDBO	CDS/HGS using SEMS	64 vs. 61	93.8 vs. 90.2	90.0 vs. 94.5	6.3 vs. 19.7*	0 vs. 14.8*	5 vs. 11* (median)
Park et al., 2018	MBO	CDS using SEMS	15 vs. 15	100 vs. 92.8	92.8 vs. 100	0 vs. 0	0 vs. 0	43 vs. 31 (mean)
Chen et al., 2023	MDBO (borderline–unresectable)	CDS using LAMS	73 vs. 71	90.4 vs. 83.1	84.9 vs. 85.9	12.3 vs. 12.7	0 vs. 5.6	14.0 vs. 23.1* (mean)
Teoh et al., 2023	Unresectable MDBO	CDS using LAMS	79 vs. 76	96.2 vs. 76.3*	93.7 vs. 90.8	16.5 vs. 17.1	0 vs. 2.6	10 vs. 25* (median)

Abbreviations: CDS, choledochoduodenostomy; HGS, hepaticogastrostomy; LAMS, lumen‐apposing metallic stent; MBO, malignant biliary obstruction; MDBO, malignant distal biliary obstruction; PEP, post‐ERCP pancreatitis.

* Statistically significant difference.

#### 3.1.1 Clinical Question 1: Can EUS‐BD Be a Primary Biliary Drainage Method for Unresectable MDBO?

Consensus statement: EUS‐BD is not considered a standard biliary drainage method for unresectable MDBO at present. EUS‐BD for unresectable MDBO is proposed for use in selected situations, such as in patients with concomitant duodenal obstruction, patients at high risk for post‐ERCP pancreatitis, or those in whom ERCP‐BD failed (*Strongly agree 85.7%, partially agree 14.3%*).

The indications and procedural practices of EUS‐BD vary considerably among countries due to differences in healthcare systems, reimbursement policies, and device availability. In the United States, there is currently no dedicated billing code for EUS‐BD, and thus reimbursement is not available—representing a major barrier to the widespread adoption of EUS‐BD. Regarding stent selection, a lumen‐apposing metal stent (LAMS; e.g., Axios, Boston Scientific, Marlborough, MA, USA) can be used in Western countries for EUS‐BD, as well as traditional tubular stents. A LAMS with a one‐step delivery system allows efficient stent deployment, which is a major technical advantage. However, compared with tubular‐type metallic stents, LAMS carry a risk of inadequate drainage after biliary decompression, as the contralateral flange may contact the bile duct wall when the duct collapses. In such cases, placement of a coaxial double‐pigtail plastic stent may help prevent adverse events, including occlusion due to food reflux. Furthermore, it remains unclear whether the results of randomized controlled trials comparing EUS‐BD and ERCP‐BD can be generalized to real‐world clinical practice, as the participating endoscopists were highly experienced in both procedures. For instance, successful stent placement during EUS‐CDS using the Axios stent, as defined in the RCT inclusion criteria, is generally technically challenging when the bile duct diameter is less than 15 mm. Safety is another critical concern. Although published data suggest that the overall adverse event rates of EUS‐BD and ERCP‐BD are comparable, EUS‐BD carries the risk of bile peritonitis, which can potentially be fatal [12]. Severe, even lethal, adverse events have been reported after EUS‐BD was performed by inexperienced endoscopists [13]. Similarly, ERCP is associated with serious adverse events, including post‐ERCP pancreatitis, which can be severe and occasionally life‐threatening. Thus, both procedures have inherent risks of major adverse events, and a balanced risk–benefit assessment is essential when selecting the optimal drainage approach. In summary, EUS‐BD cannot yet be considered the standard of care for unresectable MDBO, but may be applied in selected clinical situations. First, in patients with concomitant duodenal obstruction or stenosis, transpapillary access may be difficult or impossible, making EUS‐BD a practical alternative. Second, in patients at high risk for post‐ERCP pancreatitis (e.g., non‐dilated pancreatic duct, or a history of PEP, or anticipated difficult biliary cannulation [14]), EUS‐BD may be advantageous because it avoids papillary manipulation and has been associated with a very low incidence of pancreatitis in comparative studies as mentioned above. Third, in patients with surgically altered upper gastrointestinal anatomy, ERCP‐BD is often technically challenging, whereas EUS‐BD has demonstrated high technical success and acceptable safety in previous studies [15, 16]. Therefore, EUS‐BD may be considered in these selected clinical settings, although further evidence is needed before it can be regarded as a standard first‐line approach.

#### 3.1.2 Clinical Question 2: Which EUS‐BD Technique, CDS Versus HGS, Is Better for Unresectable MDBO?

Consensus statement: The preference between CDS and HGS for EUS‐BD in unresectable MDBO varies among countries depending on device availability, clinical experience, the degree of EUS‐BD dissemination, and anatomical constraints (*Strongly agree 85.7%, partially agree 14.3%*).

Regarding the choice between CDS and HGS for EUS‐BD in unresectable MDBO, preferences differ among countries depending on device availability, operator experience, and anatomical constraints, such as the ability to access the duodenum. Therefore, the anatomical indications for EUS‐CDS are generally more limited than those for EUS‐HGS, whereas HGS can be performed independently of duodenal access and is applicable in a broader range of clinical scenarios. A few RCTs comparing HGS with CDS in the management of unresectable MDBO have shown comparable efficacy and safety, as summarized in Table 2 [17, 18]. Furthermore, a recent meta‐analysis that included nine nonrandomized studies and two randomized controlled trials involving 537 patients (225 EUS‐HGS and 312 EUS‐CDS) demonstrated no significant difference in either technical (OR, 0.83; 95% CI, 0.41–1.68; *I* ^2^ = 0%) or clinical success rates (OR, 0.96; 95% CI, 0.51–1.81; *I* ^2^ = 9.94%) between the two approaches [19]. Recently, a retrospective study comparing EUS‐CDS and EUS‐guided gallbladder drainage (EUS‐GBD) as a first‐line approach for MDBO has been reported, suggesting that EUS‐GBD may also be a potential alternative in selected patients [20]. However, its role in this setting remains to be further established. In the United States, few endoscopists are familiar with HGS, and CDS using the Axios system is the predominant approach. In India, CDS is generally preferred; however, HGS may be selected in cases with duodenal obstruction, depending on the clinical situation. In Europe (particularly France), unpublished data suggest that CDS with LAMS may be associated with food impaction leading to stent dysfunction, highlighting the need for stents specifically designed for CDS. In contrast, in Korea, Taiwan, and Japan, HGS has become the main EUS‐BD approach owing to the widespread adoption of the technique and the availability of dedicated accessories such as 0.018‐in. guidewires compatible with 22‐gauge needles, which allow safer and more stable procedures. From a technical and mechanistic perspective, CDS and HGS differ in their drainage routes. CDS enables antegrade bile drainage into the duodenum, which is considered more physiological. In contrast, HGS enables retrograde bile drainage into the stomach. These differences may influence stent patency, the risk of adverse events, and overall clinical outcomes. Therefore, the choice between CDS and HGS should be individualized based not only on healthcare environments, device availability, and operator experience but also on anatomical conditions and disease characteristics. Well‐designed randomized controlled trials directly comparing CDS and HGS are warranted to better define their optimal indications and clinical roles. To facilitate a clinically meaningful comparison between CDS and HGS, a summary of their technical characteristics, anatomical applicability, and clinical considerations is provided in Table 3.

**TABLE 2:** Randomized controlled studies comparing EUS‐CDS versus EUS‐HGS.

Study (author, year)	Subject	Details of EUS‐BD	CDS versus HGS				
			Number of patients, *n*	Technical success, %	Clinical success, %	Adverse event, %	Procedure time, min
Artifon et al., 2015	Unresectable MDBO	CDS/HGS using PCMS	24 vs. 25	91.7 vs. 96.0	77.3 vs. 91.7	12.5 vs. 25.0	48.8 vs. 47.8 (mean)
Minaga et al., 2019	Unresectable MDBO	CDS/HGS using PCMS	23 vs. 24	82.6 vs. 87.5*	95.7 vs. 100	8.7 vs. 8.3	30.5 vs. 39.2 (mean)

Abbreviations: CDS, choledochoduodenostomy; EUS‐BD, endoscopic ultrasound‐guided biliary drainage; HGS, hepaticogastrostomy; MDBO, malignant distal biliary obstruction; PCMS, partially covered metallic stent.

* Statistically significant difference.

**TABLE 3:** Comparison of technical and clinical features between EUS‐CDS and EUS‐HGS.

	EUS‐CDS	EUS‐HGS
Drainage route	Antegrade drainage into the duodenum	Retrograde drainage into the stomach
Physiological aspect	More physiological biliary drainage	Less physiological biliary drainage
Access requirement	Requires duodenal access	Does not require duodenal access
Feasibility in duodenal obstruction	Limited/often not feasible	Feasible
Typical indication	Distal biliary obstruction with accessible duodenum	Duodenal obstruction or difficult duodenal access
Technical characteristics	LAMS enables one‐step deployment	Technically more demanding; multi‐step procedure
Common stents	LAMS or tubular CSEMS	PCSEMS or dedicated plastic stents
Advantages	Physiological drainage; shorter procedure time in selected cases	Applicable regardless of duodenal access
Limitations	Not suitable in duodenal obstruction	Non‐physiological drainage; technical complexity

Abbreviations: CDS, choledochoduodenostomy; CSEMS, covered self‐expandable metallic stent; HGS, hepaticogastrostomy; LAMS, lumen‐apposing metallic stent; PCSEMS, partially covered self‐expandable metallic stent.

#### 3.1.3 Clinical Question 3: How Can EUS‐BD Become the Primary Biliary Drainage?

Consensus statement: The development of dedicated devices and delivery systems as well as the establishment of standardized techniques to simplify and secure the procedure are required to establish EUS‐BD as a primary drainage option for unresectable MDBO. The training of expert endoscopists skilled in both ERCP and interventional EUS is essential for the adoption of EUS‐BD in clinical practice (*Strongly agree 100%*).

To establish EUS‐BD as a primary drainage method for unresectable MDBO, further improvements in device design and refinements in procedural techniques are required to simplify the multi‐step process and enhance procedural safety. EUS‐BD involves multiple steps, including bile duct puncture, guidewire placement, tract dilation, and stent deployment. The Axios system enables one‐step stent deployment; however, the stent itself was not specifically designed for EUS‐BD. Therefore, the development of dedicated biliary drainage stents and simplified delivery systems optimized for EUS‐BD is warranted. In recent years, various dedicated devices for EUS‐BD have been developed, including tract dilation devices and stents designed to improve procedural safety and simplify the procedure. However, the availability of these devices varies considerably among countries, and it is beyond the scope of this manuscript to comprehensively review all currently available devices. From a technical standpoint, interventional radiologists often use a safety wire to ensure procedural safety. A similar concept can be applied to EUS‐BD, where the establishment of standardized, reproducible, and safety‐oriented procedural techniques is essential. In this context, the double guidewire technique has been reported as a potential approach—not only serving as a safety wire but also helping to straighten the tract, thereby stabilizing subsequent device insertion and stent placement [21]. However, the double guidewire technique should be considered as one of several technical options rather than a standardized method, as it may increase procedural complexity and prolong tract manipulation, which could theoretically increase the risk of bile leakage. Therefore, further evidence is needed to clarify its overall safety and clinical benefit. Because EUS‐BD carries the risk of severe adverse events such as bile peritonitis, the procedure should be performed by expert endoscopists skilled in both ERCP and interventional EUS, and the training of such experts is essential for establishing EUS‐BD as a standard procedure. From a health economics perspective, cost considerations are also important when evaluating the role of EUS‐BD as a primary drainage method. In particular, lumen‐apposing metal stents (LAMS) are relatively expensive compared with conventional transpapillary stents, which may limit their widespread use in certain healthcare settings. In addition, the learning curve of EUS‐BD should be taken into account. The procedure is technically demanding, and clinical outcomes may vary depending on operator experience. Therefore, careful case selection and appropriate training are essential, particularly in centers with limited experience. Finally, the establishment of appropriate procedural billing codes and reimbursement systems is also essential for the widespread adoption of EUS‐BD in many countries.

### 3.2 Topic 2: EUS‐BD for Biliary Diseases in Patients With SAA

In the management of biliary diseases in patients with SAA, endoscopic access to the biliary system is technically challenging due to anatomical complexity, and percutaneous approaches have traditionally been performed. The development of balloon endoscopy (BE) enables reliable insertion of the scope to the biliary orifice or the anastomosis, and the clinical usefulness of BE‐assisted ERCP (BE‐ERCP) has been reported [22, 23]. However, the procedure remains technically demanding and time‐consuming, particularly in terms of endoscope insertion and biliary cannulation. As a novel treatment option, EUS‐BD has been introduced, and its efficacy in the management of biliary disorders has been increasingly recognized in patients with SAA. In addition, EUS‐directed ERCP techniques, such as EUS‐directed transgastric ERCP (EDGE), have emerged as alternative approaches for biliary intervention in patients with surgically altered anatomy, expanding the therapeutic options in this setting [24]. Comparative prospective and retrospective studies have evaluated EUS‐BD and BE‐ERCP for malignant biliary obstruction (MBO), including both hilar and distal biliary obstruction, and bile duct stones (BDS) and benign biliary strictures (BBS) (Table 4) [16, 25, 26, 27, 28, 29]. In patients with MBO, EUS‐BD consistently demonstrated higher technical success rates with comparable clinical outcomes and adverse event rates, while procedure time was significantly shorter with EUS‐BD. For the treatment of BDS, both BE‐ERCP and EUS‐AG approaches have shown similar efficacy and safety; however, EUS‐AG was superior in terms of procedural efficiency, whereas BE‐ERCP tended to achieve a higher rate of complete stone clearance. In BBS, trans‐ESCR procedures, including balloon dilation and stent placement, have been retrospectively evaluated, demonstrating favorable outcomes. In BBS, mainly hepaticojejunal anastomotic strictures, high endoscopic access rates to the anastomotic site and high procedure success rates for balloon dilation and stent placement by BE‐ERCP have been reported [30, 31]. However, balloon dilation alone results in a high recurrence rate, requiring multiple plastic stenting or metal stenting procedures [32, 33, 34]. Therefore, many cases require repeated procedures. From the perspective of re‐intervention, EUS‐BD may offer potential advantages, as access to the biliary system can be achieved through the established EUS‐guided created route without the need for repeated deep scope insertion. This may facilitate subsequent interventions and reduce procedural burden in selected patients. Recently, the favorable outcomes of treatments for anastomotic strictures, including balloon dilation and stenting via the route created by EUS‐BD, have been reported; however, no studies have directly compared BE‐ERCP with EUS‐BD [35, 36, 37].

**TABLE 4:** Retrospective studies comparing EUS‐BD versus ERCP‐BD for biliary diseases in patients with surgically altered anatomy.

Study (author, year)	Type of study	Subject	EUS‐BD versus ERCP‐BD					
			Number of patients, *n*	Technical success, %	Clinical success, %	Adverse event, %	PEP (%)	Procedure time, min
Khashab et al., 2016	Retrospective	Biliary obstruction	49 vs. 49	98.0 vs. 65.3*	88.0 vs. 59.1*	4.1 vs. 20.4*	0 vs. 0.2	55.0 vs. 95.7* (mean)
Takasaki et al., 2021	Retrospective	BDS	23 vs. 31	87.0 vs. 64.5	73.9 vs. 67.7	17.4 vs. 12.9	0 vs. 9.7	51.9 vs. 72.6* (mean)
Iwashita et al., 2023	Retrospective	BDS	23 vs. 96	65.2 vs. 69.8	NA	17.4 vs. 7.3	0 vs. 3.1	42 vs. 64* (median)
Hakuta et al., 2024	Retrospective	MBO	32 vs. 118	94 vs. 70*	90 vs. 84	22 vs. 14	0 vs. 5.9	45 vs. 60 (median)
Sato et al., 2024	Retrospective	BDS	59 vs. 588	83.1 vs. 83.7	67.8 vs. 78.1	18.6 vs. 10.2	0 vs. 3.9	61.5 vs. 90* (median)
Itonaga et al., 2025	Prospective	MBO	44 vs. 54	95 vs. 74*	93 vs. 83	16 vs. 9	5.5 vs. 2.2	45.7 vs. 83.6* (mean)
Shibuki et al., 2025	Retrospective	MBO	29 vs. 41	100 vs. 68.3*	93.1 vs. 96.4	20.6 vs. 17.1	6.8 vs. 4.9	23 vs. 50* (median)

Abbreviations: BDS, bile duct stone; MBO, malignant biliary obstruction; PEP, post‐ERCP pancreatitis.

* Statistically significant difference.

#### 3.2.1 Clinical Question 1: Which Approach Is Preferable for Malignant Bile Duct Obstruction in Patients With Surgically Altered Anatomy, the BE‐ERCP Approach or the EUS‐BD Approach?

Consensus statement: When the safety of the procedure is ensured at institutions with experienced experts, EUS‐BD can be considered as a primary drainage option, taking into account patient characteristics, anatomical factors, and institutional experience (*Strongly agree 61.5%, partially agree 30.8%, strongly disagree 7.7%*).

BE‐ERCP approach for malignant biliary obstruction in patients with SAA is generally considered a safer approach with a lower adverse event rate than EUS‐BD. However, the procedure is technically challenging, with longer procedural duration and lower success rates compared with EUS‐BD. The prolonged procedural time results in physical burdens to both the endoscopist and the patient, and prolonged anesthesia may also increase procedural risk. Many facilities lack a short‐type, therapeutic channel balloon endoscope, which allows the use of traditional ERCP devices. Long‐type balloon endoscopes further increase the technical difficulty due to limitations on the application of devices that fit the smaller size and longer accessory channel of balloon endoscopes. There is a technique to change endoscopes after inserting a balloon endoscope to the papilla or anastomotic site, but it is technically demanding [38]. On the other hand, with the widespread application of EUS‐BD techniques and the development of various devices, the success rate of EUS‐BD has improved, and the adverse event rate has decreased [39]. Therefore, while BE‐ERCP remains the first‐line approach in many Japanese institutions, the choice of drainage modality should be individualized. In cases with limited tumor invasion and a short afferent loop in which the BE‐ERCP approach is expected to be technically straightforward, BE‐ERCP is often preferred, particularly in high‐volume BE‐ERCP centers. In contrast, in institutions with experienced EUS‐BD experts and established safety protocols, EUS‐BD may be considered as a primary drainage option for malignant biliary obstruction in patients with SAA. However, EUS‐BD also has important limitations. In particular, it may not be feasible in cases without dilation of the left intrahepatic bile duct, especially when only the right hepatic duct is dilated. In addition, in patients with separate right and left hepaticojejunostomies, conventional EUS transhepatic BD may not provide adequate drainage of the right hepatic duct. Therefore, careful anatomical assessment is essential when selecting the optimal drainage approach. For cases where the EUS‐BD approach is difficult, either BE‐ERCP or percutaneous transhepatic biliary drainage (PTBD) may be chosen based on institutional expertise.

#### 3.2.2 Clinical Question 2: What Is the Commonly Performed EUS‐BD Procedure for Malignant Bile Duct Obstruction in Patients With Surgically Altered Anatomy?

Consensus statement: EUS‐HGS using a partially covered metallic stent is commonly performed in patients with surgically altered anatomy. EUS‐HGS using a dedicated plastic stent can be a useful option, though it is available only in a limited number of countries (*Strongly agree 64.3%, partially agree 28.6%*).

In patients with SAA, EUS‐CDS is anatomically difficult or impossible to perform, and EUS‐HGS, including hepaticojejunostomy in patients with gastrectomy, is selected. A partially covered metallic stent (PCMS), where 1–2 cm at the hepatic end or half of the stent is uncovered, is most commonly used worldwide when performing HGS [40, 41]. The uncovered part of the hepatic end prevents obstruction of the branch bile ducts and may help reduce the risk of stent migration at the liver side due to anchoring of the uncovered portion within the intrahepatic bile duct. However, early stent dislocation at the liver side has also been reported, and therefore its protective effect should be interpreted with caution. In recent years, PCMS with an anti‐migration function at the gastric end has also been produced, further reducing migration risk and improving safety [42, 43]. Therefore, PCMS are widely used for EUS‐HGS. A disadvantage of PCMS is that stent removal becomes difficult after long‐term placement, which can complicate re‐intervention and is not recommended for the treatment of benign conditions. In contrast, fully covered metallic stents (FCMS) allow easier removability, making them advantageous in situations where future re‐intervention is anticipated [44, 45], although the risk of stent migration and cholangitis or hepatic abscess due to obstruction of side branch ducts should be considered. Additionally, if available, EUS‐HGS using a dedicated single‐pigtail PS is also a useful option [46]. It can be inserted with minimal tract dilation due to its good insertability, and it can reduce bile leakage compared with conventional PSs. Combined antegrade stenting and HGS improves drainage efficacy and prolongs stent patency [47]. Given the heterogeneity of clinical scenarios, stent selection should be individualized, and further prospective studies are needed to better define the optimal stent choice in EUS‐HGS.

#### 3.2.3 Clinical Question 3: Which Approach Is Preferable for Benign Biliary Disease in Patients With Surgically Altered Anatomy, the BE‐ERCP Approach or the EUS‐BD Approach?

Consensus statement: The optimal approach is selected based on the expertise of each institution, available devices, intestinal reconstruction method, and treatment strategy for benign disease (*Strongly agree 78.6%, partially agree 21.4%*).

In the setting of benign biliary obstruction, intrahepatic ductal dilation is less pronounced than in cases of malignant biliary obstruction. Additionally, in some cases, the bile duct wall may be rigid due to recurrent cholangitis, making EUS‐BD more challenging [48]. Furthermore, subsequent treatment for bile duct stones and anastomotic strictures must also be considered in addition to drainage. Antegrade treatment via the EUS‐BD route is often challenging in many cases. On the other hand, BE‐ERCP can also be technically demanding, particularly in patients with Roux‐en‐Y reconstruction and in those with an intact papilla, in whom scope insertion, selective biliary cannulation, and subsequent therapeutic interventions are often difficult [27, 28]. BE‐ERCP has been reported to yield a higher overall treatment success rate when the papilla or anastomotic site can be successfully accessed [27]. Therefore, in institutions with high expertise in BE‐ERCP and with short‐type balloon endoscopes, BE‐ERCP is preferred as the first choice. However, if an institution lacks expertise in BE‐ERCP and has more experience with EUS‐BD, EUS‐BD may be acceptable as the first choice. When both approaches are difficult due to the institution's situation or other factors, the percutaneous approach may be selected. When selecting a treatment approach, intestinal reconstruction methods must also be considered. In cases after Whipple surgery or gastrectomy, the afferent limb is not long, so reaching the papilla or anastomosis with an enteroscope can be easy. However, in patients with Roux‐en‐Y gastric bypass surgery, insertion is not easy because the afferent limb is typically long. The optimal treatment approach also depends on the conditions of bile duct stones and anastomotic strictures. While cholangioscopy‐assisted lithotripsy via EUS‐BD can be effective in large and hard stones, most stone extraction devices are available for use with the short‐type enteroscope [49]. In cases of severe anastomotic stricture with a high risk of recurrence requiring repeated interventions, a novel approach called EUS‐directed transgastric endoscopic retrograde cholangiopancreatography (EDGE) is notable [50]. At the present time, no single approach is preferred. The selection should be based on a comprehensive consideration of each institution's expertise, available devices, intestinal reconstruction methods, and subsequent treatment strategies for benign conditions following drainage.

#### 3.2.4 Clinical Question 4: What Is the Preferred EUS‐BD Procedure for Benign Biliary Disease in Patients With Surgically Altered Anatomy?

Consensus statement: In patients with surgically altered anatomy and benign biliary disease, the choice of EUS‐BD procedure should be individualized based on the availability of stents, tract dilation devices, need for subsequent antegrade therapy, and long‐term strategies. At present, EUS‐HGS using FCMS with anti‐migration function, or a dedicated PS with removability, may be considered in selected cases (*Strongly agree 76.9%, partially agree 23.1%*).

In benign cases, considering additional antegrade treatment and long‐term treatment strategies, removability is mandatory for stents placed via the EUS‐HGS route including hepaticojejunostomy in patients with gastrectomy. Therefore, PS or FCMS are preferred over PCMS. The use of FCMS raises concerns about serious adverse events due to stent migration before tract maturation, but in recent years, FCMS with anti‐migration function has been produced with improved safety [51]. This also has the advantage of creating a large access route to the bile duct, thereby providing more options for subsequent antegrade procedures. On the other hand, a dedicated PS is a useful option with an extremely low risk of stent migration or dislodgement, and a lower risk of adverse events such as cholangitis or liver abscess due to bile duct branch obstruction. However, such dedicated cPSs are not widely available. Minimal but effective dilation devices such as bougie and drill dilators are being produced, especially in Japan, but these devices are not currently widely available [52]. A cautery dilator is widely used as a useful tract dilation device, although it carries a slightly higher risk of bleeding than mechanical tract dilation devices. Strategies for antegrade treatment should be selected on a case‐by‐case basis. For small stones, sequential antegrade treatment may be performed immediately following EUS‐BD procedures. In some cases of large stones, cholangioscopy‐assisted lithotripsy via the EUS‐HGS route may be required after tract maturation, typically more than 2 weeks after the initial procedure. In cases with severe anastomotic strictures, multi‐stenting across the anastomotic site while dilating the EUS‐HGS route may be useful for preventing stricture recurrence. Given the heterogeneity of disease conditions, treatment strategies should be individualized. Further prospective studies are required to evaluate the optimal treatment strategy and stent choice.

### 3.3 Topic 3: Japanese Proposal for the Classification and Terminology of Interventional EUS

During this EUS‐BD consensus meeting, two specific aspects of the classification and terminology were discussed:

1. The terminology of drainage and anastomosis
2. The concept of trans‐EUS‐guided created route (trans‐ESCR) procedures

#### 3.3.1 EUS‐Guided Drainage and Anastomosis

First, the terms *drainage* and *anastomosis* were discussed. The term *drainage* is widely used in ERCP, which utilizes physiological routes. However, in interventional EUS, the created route is non‐physiological and connects two organs directly—essentially forming an anastomosis. Therefore, in EUS‐BD, the procedure should be termed anastomosis rather than drainage. In contrast, EUS‐guided drainage of acquired fluid collections, such as pancreatic fluid collections or abscesses, leads to disappearance of the collection; hence, such procedures are appropriately referred to as drainage, not anastomosis. Although the stents used in interventional EUS resemble those in ERCP, their placement sites are quite different. In ERCP, stents are positioned across a stricture or obstruction, while in interventional EUS, they are placed through the gastrointestinal wall to create an anastomosis or drainage route. Therefore, we propose the term transluminal drainage/anastomosis stents (T‐DAS) for stents used in interventional EUS. A basic principle for naming EUS‐guided anastomoses is also proposed: the order of organs in the term should follow the direction of fluid or content flow, as traditionally used in surgical nomenclature. For example, in EUS‐CDS, bile flows from the common bile duct (*choledocho‐*) into the duodenum (*‐duodeno‐*) through the anastomosis (*‐stomy*).

#### 3.3.2 Trans‐ESCR Procedures

Recently, various endoscopic procedures have been performed through routes created by interventional EUS procedures—for instance, direct endoscopic necrosectomy following EUS‐guided drainage of pancreatic fluid collection, or antegrade stone extraction following EUS‐HGS.

To classify such procedures, we have proposed the term ESCR (Endosonographically Created Route), and have defined procedures performed via this route as trans‐ESCR procedures. As the number of trans‐ESCR procedures continues to grow, it is important to establish consistent principles for describing them. For example, ERCP performed through an EUS‐guided gastrogastrostomy in patients with gastric bypass is currently termed EDGE. However, this terminology cannot be easily applied to similar procedures. According to our principle, EDGE can be expressed more precisely as ERCP following EUS‐guided gastrogastrostomy with LAMS.

#### 3.3.3 Comments by the Panel Members

Following the presentation, several points and concerns were raised. The proposed terminology for interventional EUS is logical, though perhaps not intuitive. In interventional radiology, for example, the term PTBD (Percutaneous Transhepatic Biliary Drainage) is well established. Following this model, ETBD (Endoscopic Transhepatic Biliary Drainage) might seem more intuitive for a procedure performed through the liver. However, it is worth noting that the term ERCP also became established only after extensive debate in the 1970s. In Japan, the procedure was originally called ECPG (Endoscopic Cholangiopancreatography), but “ERCP” is now accepted internationally, including in Japan. Because the proposed terminology is logical, it may gradually gain acceptance, though it will likely require time. Some international attendees found the term “ESCR” confusing, as they were unfamiliar with it. However, this terminology is already being used increasingly in Japan and has also been proposed in Taiwan. With wider adoption, it may become more familiar and accepted globally. Another potential advantage of adopting standardized terminology lies in facilitating reimbursement for newly developed procedures. In Japan, for instance, reimbursement rates differ for certain stents depending on whether they are used in ERCP or EUS‐BD. A unified terminology could help justify and standardize such distinctions. In summary, while the proposed terminology for interventional EUS is logical, it may lack intuitive clarity—likely because it was developed primarily among Japanese experts. Nevertheless, there is general agreement on the need for a unified global terminology in this evolving field. Continued international collaboration and discussion are encouraged to refine and harmonize these concepts.

## Author Contributions

Takuji Iwashita, Shuntaro Mukai, and Yousuke Nakai wrote the manuscript. Takuji Iwashita, Shuntaro Mukai, Yousuke Nakai, Shomei Ryozawa, Akio Katanuma, Ichiro Yasuda, Atsushi Irisawa, Sang‐Soo Lee, Jong Ho Moon, Hsui‐Po Wang, Sundeep Lakhtakia, Marc Giovannini, Todd H. Baron, and Takao Itoi participated in the meeting and Delphi process. Takao Itoi supervised the manuscript.

## Funding

The authors have nothing to report.

## Conflicts of Interest

Shuntaro Mukai received honoraria for lectures from Gadelius Medical Co. Todd H. Baron is Speaker and Consultant for Boston Scientific, Cook Endoscopy, Olympus America, WL Gore, GIE Medical, and ASAHI Intecc Medical. Other authors declare no conflicts of interest related to this article.

## Data Availability

The data that support the findings of this study are available on request from the corresponding author. The data are not publicly available due to privacy or ethical restrictions.
